# Association of Hypertrophic Obstructive Cardiomyopathy With Outcomes Following Transcatheter Aortic Valve Replacement

**DOI:** 10.1001/jamanetworkopen.2019.21669

**Published:** 2020-02-21

**Authors:** Dhrubajyoti Bandyopadhyay, Sandipan Chakraborty, Birendra Amgai, Samir R. Kapadia, Eugene Braunwald, Srihari S. Naidu, Ankur Kalra

**Affiliations:** 1 Icahn School of Medicine at Mount Sinai, St Luke’s Roosevelt Hospital, New York, New York; 2 Interfaith Medical Center, Brooklyn, New York; 3 Heart and Vascular Institute, Department of Cardiovascular Medicine, Cleveland Clinic, Cleveland, Ohio; 4 TIMI Study Group, Cardiovascular Division, Brigham and Women's Hospital, Boston, Massachusetts; 5 Westchester Medical Center and New York Medical College, Valhalla, New York

## Abstract

This cohort study investigates the association of hypertrophic obstructive cardiomyopathy with outcomes following transcatheter aortic valve replacement.

## Introduction

Hypertrophic cardiomyopathy (HCM) and valvular aortic stenosis can both present with left ventricular outflow obstruction and hypertrophy. In patients with aortic stenosis and coexisting HCM undergoing transcatheter aortic valve replacement (TAVR), this can pose a management dilemma, as one condition can modulate the effects of the other. Indeed, there has been anecdotal concern that treating the aortic valve prior to treating outflow tract obstruction may result in higher mortality due to exacerbation of subvalvular obstruction. There are limited data on optimal management or outcomes of coexisting HCM and aortic stenosis in patients who undergo TAVR. We conducted a retrospective cohort study using the National Inpatient Sample to identify the association of known HCM with outcomes following TAVR.

## Methods

We queried the 2012 to 2016 National Inpatient Sample to identify all patients (aged ≥18 years) who underwent TAVR and had coexisting HCM by using respective *International Classification of Diseases, Ninth Edition, Clinical Modification (ICD-9-CM)* or *International Statistical Classification of Diseases, Tenth Revision, Clinical Modification Procedure Coding System (ICD-10-CM/PCS)* codes. Our primary outcome was in-hospital mortality. Categorical data were presented as counts and percentages, and continuous data as means with standard deviations or standard errors. Categorical variables were analyzed using Pearson χ^2^ tests, and continuous data were analyzed using *t* tests. We generated univariable and multivariable models to study the influence of HCM on outcomes, adjusting the model for relevant baseline characteristics. All analyses in our study were weighted using provided discharge weights to produce national estimates. Stata/IC software version 15.10 (StataCorp, LLC) was used for statistical analysis. Statistical significance was set at 2-tailed *P* < .05. Given the deidentified nature of the National Inpatient Sample data, our study was exempt from approval from the institutional review boards of Mount Sinai St Luke’s West and the Cleveland Clinic. This study was conducted as per the Strengthening the Reporting of Observational Studies in Epidemiology (STROBE) reporting guideline.

## Results

A total of 100 495 patients underwent TAVR during the study period. Of these, 230 patients (0.22%) had concomitant HCM. Compared with patients without HCM, those with HCM were more likely to be women (78.3% vs 46.7%; *P* < .001) and obese (28.3% vs 15.4%; *P* = .02) but less likely to have prior coronary artery bypass grafting (0.7% vs 21.4%; *P* = .01). Baseline demographic characteristics are presented in Table 1.

**Table 1.** Baseline Demographic Characteristics of Patients Undergoing Transcatheter Aortic Valve Replacement With and Without Concomitant HCM

Characteristic	No. (%)		*P* Value
	Patients Without HCM (n = 100 265)	Patients With HCM (n = 230)	
Women	46 780 (46.7)	180 (78.3)	<.001
Age, mean (SD), y	80.72 (21.00)	80.99 (27.30)	.86
Race			
White	81 905 (87.2)	175 (83.3)	.01
Black	3935 (4.2)	20 (9.5)	
Hispanic	3850 (4.1)	5 (2.4)	
Asian	1125 (1.2)	0	
Native American	155 (0.2)	5 (2.4)	
Other	2940 (3.1)	5 (2.4)	
Charlson Comorbidity Index score			
0	11 440 (11.4)	35 (15.2)	.04
1	16 195 (16.2)	70 (30.4)	
2	19 045 (19.0)	40 (17.4)	
≥3	53 585 (53.4)	85 (37.0)	
Mean annual income in patient zip code, percentile			
0-25th	21 090 (21.4)	45 (19.6)	.67
26th-50th	24 565 (24.9)	45 (19.6)	
51st-75th	25 855 (26.2)	55 (24.4)	
76th-100th	27 115 (27.5)	80 (35.6)	
Insurance type			
Medicare	90 475 (91.5)	205 (91.1)	.87
Medicaid	1095 (1.1)	5 (2.2)	
Private	6770 (6.9)	15 (6.5)	
Uninsured	540 (0.5)	0	
Hospital characteristics			
Region			
Northeast	25 120 (25.1)	90 (39.1)	.10
Midwest	22 840 (22.8)	55 (23.9)	
South	33 590 (33.5)	50 (21.7)	
West	18 715 (18.7)	35 (15.2)	
Bed size			
Small	5165 (5.1)	15 (6.5)	.91
Medium	17 925 (17.9)	40 (17.4)	
Large	77 175 (77.0)	175 (76.1)	
Urban hospital location	99 455 (99.2)	225 (97.8)	.29
Teaching hospital	89 445 (89.2)	215 (93.5)	.34
Patient comorbidities			
Anemia (both deficiency and blood loss)	3625 (3.6)	15 (6.5)	.29
Prior stroke	11 655 (11.6)	15 (6.5)	.27
Prior myocardial infarction	13 215 (13.2)	15 (6.5)	.18
Prior percutaneous coronary intervention	12 985 (13.0)	35 (15.2)	.64
Prior coronary artery bypass surgery	21 465 (21.4)	15 (6.5)	.01
Pulmonary hypertension	21 500 (21.4)	75 (32.6)	.08
Coronary artery disease and coronary artery disease equivalent	62 005 (61.8)	115 (50.0)	.09
Hypertension	46 880 (46.8)	95 (41.3)	.45
Obesity	15 400 (15.4)	65 (28.3)	.02
Dyslipidemia	65 370 (65.2)	140 (60.9)	.53
Peripheral vascular disease	18 190 (18.1)	30 (13.04)	.36
Chronic lung disease	32 125 (32.0)	50 (21.7)	.12
Congestive heart failure	64 370 (64.2)	135 (58.7)	.41
Diabetes with and without complications	36 005 (35.9)	60 (26.1)	.14
Chronic kidney disease	36 090 (36.0)	75 (32.6)	.62

Abbreviation: HCM, hypertrophic cardiomyopathy.

Patients with HCM had greater incidence of in-hospital mortality (18.6% vs 2.91% for non-HCM; adjusted odds ratio [aOR], 7.33; 95% CI, 3.26-16.44; *P* < .001), aortic dissection (2.33% vs 0.39%; aOR, 10.50; 95% CI, 2.53-43.53; *P* = .001), acute kidney injury (23.26% vs 12.24%; aOR, 2.62; 95% CI, 1.21-5.66; *P* = .01), and postoperative shock (16.28% vs 3.43%; aOR, 4.67; 95% CI, 1.97-11.05; *P* < .001) (Table 2). There were no differences in terms of vasopressor use, pacemaker requirement, vascular injury, major bleeding requiring transfusion, and respiratory failure between the 2 groups.

**Table 2.** Association of HCM With In-Hospital Outcomes

Outcome	Incidence, %		Unadjusted		Adjusted^a^	
	HCM	Non-HCM	OR (95% CI)	*P* Value	OR (95% CI)	*P* Value
In-hospital mortality	18.6	2.9	7.61 (3.52-16.44)	<.001	7.33 (3.26-16.44)	<.001
Aortic dissection	2.3	0.4	6.02 (0.82-43.71)	.07	10.50 (2.53-43.53)	.001
Acute kidney injury	23.3	12.2	2.17 (1.06-4.45)	.03	2.62 (1.21-5.66)	.01
Postoperative shock	16.3	3.4	5.48 (2.42-12.38)	<.001	4.67 (1.97-11.05)	<.001
Vasopressor use	4.7	1.9	2.46 (0.59-10.29)	.21	3.14 (0.97-10.19)	.06
Pacemaker requirement	2.3	7.0	0.31 (0.04-2.31)	.25	0.32 (0.004-2.36)	.26
Complete heart block	4.7	10.4	0.42 (0.10-1.75)	.23	0.60 (0.18-1.95)	.40
Respiratory failure	14.0	10.2	1.43 (0.60-3.39)	.41	1.8 (0.65-3.89)	.49
Mechanical ventilation	4.7	2.4	1.98 (0.47-8.26)	.34	1.45 (0.33-6.40)	.61
Major bleeding requiring transfusion	18.6	18.1	1.03 (0.48-2.22)	.92	1.02 (0.49-2.12)	.94
Vascular injury	2.3	0.7	3.43 (0.47-25.10)	.22	4.17 (0.57-22.1)	.89

Abbreviations: HCM, hypertrophic cardiomyopathy; OR, odds ratio.

^a^ Variables adjusted were age, female sex, history of stroke, history of myocardial infarction, history of percutaneous coronary intervention, history of coronary artery bypass grafting, pulmonary hypertension, coronary artery disease, hypertension, obesity, dyslipidemia, peripheral vascular disease, chronic lung disease, diabetes, congestive heart failure, chronic kidney disease, anemia, Charlson Comorbidity Index category, elective procedure, insurance, teaching status of the hospital, and location of the hospital.

## Discussion

In this large, nationally representative study, we found that presence of HCM in patients undergoing TAVR was associated with markedly increased in-hospital mortality and complications. Current data on the association between HCM and postprocedural outcomes after TAVR are limited to small case reports and case series, which have described acute hemodynamic compromise after valve deployment.^1,2,3^ These cases highlight the difficulty in determining the contributions of each of the 2 obstructive lesions, and that provocable gradients might best be treated prior to TAVR to avoid the so-called suicide ventricle due to rapid removal of the afterload of aortic stenosis.^1,2,3^ The increased mortality observed in our study is likely attributable to unanticipated postprocedural hemodynamic compromise caused by unmasking of left ventricular outflow tract obstruction due to HCM, as previously reported.^1,3^ The higher rates of acute kidney injury may be associated with postprocedural hemodynamic alterations and volume depletion due to the use of diuretics to optimize volume status prior to TAVR. The increased use of vasoactive medications in the HCM cohort in our study was also described by others^2^; these may be needed to maintain afterload to counter the left ventricular outflow tract obstruction caused by HCM, as well as to treat the development of cardiogenic shock. The main limitations of our study were retrospective observational study design, a smaller number of patients in the HCM group, possibility of coding errors, and lack of granular information on hemodynamic data. Nevertheless, our results highlight a need for further work to investigate the impact of HCM, if any, on long-term outcomes following TAVR.

In this study, concomitant HCM was associated with substantially worse in-hospital outcomes, including cardiogenic shock, renal failure, and death, in patients undergoing TAVR.
